# Coinfection of cytomegalovirus and strongyloidiasis presenting as massive gastrointestinal bleeding in an immunocompromised host: a case report

**DOI:** 10.3389/fmed.2024.1438689

**Published:** 2024-11-20

**Authors:** Thanh Nguyen Huu, Hoai Mai Thu, Khoa Quy, Mai Vo Thanh, Nga Dinh Thi, Quang Pham Minh Ngoc, Tuyet Duong Thi, Binh Nguyen Canh, Tung Nguyen Lam, Ky Thai Doan

**Affiliations:** ^1^College of Health Sciences, VinUniversity, Hanoi, Vietnam; ^2^Department of Gastroenterology, 108 Military Central Hospital, Hanoi, Vietnam

**Keywords:** cytomegalovirus, strongyloidiasis, coinfection, gastrointestinal bleeding, immunocompromised host, a case report

## Abstract

Cytomegalovirus (CMV) infection is an opportunistic disease in immunocompromised patients that may appear without symptoms, with constitutional symptoms, or as a tissue-invasive disease. *Strongyloides stercoralis* infection often manifests with non-specific symptoms; however, it can lead to severe malabsorption and extraintestinal dissemination by accelerated autoinfection. The coinfection of CMV and *Strongyloides stercoralis* has rarely been reported, particularly with solely severe gastrointestinal bleeding. A 29-year-old female patient with a history of nephrotic syndrome treated with long-term corticosteroid and poorly controlled type 2 diabetes presented with a 20-day history of persistent epigastric pain, diarrhea, and significant weight loss. At the hospitalization, the patient appeared to have persistent gastrointestinal bleeding, leading to hypovolemic shock and diabetic ketoacidosis. *Strongyloides stercoralis* was detected by the duodenal biopsy results, and the polymerase chain reaction of these samples was positive for CMV. The patient underwent upper endoscopy four times to control the bleeding and was treated with ivermectin and ganciclovir. The patient improved gradually and was discharged after 23 days of hospitalization. The coinfection of CMV and *Strongyloides stercoralis* causing massive gastrointestinal (GI) bleeding has been rarely reported. To the best of our knowledge, this is also the first case of coinfection of these pathogens in an immunocompromised patient complicated with hypovolemic shock caused by GI bleeding and diabetic ketoacidosis. Clinicians should have a high index of suspicion and test simultaneously CMV and *Strongyloides stercoralis* in patients with immunosuppression, other risk factors, or unexplained gastrointestinal symptoms.

## 1 Introduction

Cytomegalovirus (CMV) infection is a common opportunistic pathogen in immunocompromised patients that can lead to severe organ damage (1). CMV infection may appear without symptoms, with constitutional symptoms, or as a tissue-invasive disease. Gastrointestinal CMV infection presents with dysphagia, abdominal discomfort, diarrhea, and bleeding in the upper or lower gastrointestinal (GI) tract and is associated with 30% of tissue-invasive diseases among immunocompetent patients (2, 3).

Strongyloidiasis is an infection caused by *Strongyloides stercoralis*, which is prevalent in tropical and subtropical areas (4). Its larvae can get ingested into the gastrointestinal system and lay eggs in the mucous lining of the small intestine, particularly in the duodenum and upper jejunum (5). *S. stercoralis* infection often presents with a wide spectrum of clinical symptoms, ranging from asymptomatic to an acute gastrointestinal episode and life-threatening disseminated infections (6). Moreover, it can induce a severe hyperinfection condition, particularly in those with compromised immune systems and those with long-term corticosteroid use as it leads to an accelerated autoinfection cycle (5).

There are a few case reports about the coinfection of CMV and *Strongyloides stercoralis* in immunocompromised individuals, including HIV, diabetes, or hematological malignancies (7–9). However, these conditions have been rarely reported with severe GI bleeding. We report a rare case of severe GI bleeding resulting in hypovolemic shock caused by the coinfection with CMV and *Strongyloides stercoralis* in compromised patients. To the best of our knowledge, this is the first case reported.

## 2 Case presentation

A 29-year-old female patient with a history of nephrotic syndrome and type 2 diabetes presented with a 20-day history of persistent epigastric pain and diarrhea. She was diagnosed with nephrotic syndrome 4 years ago, which was treated with methylprednisolone frequently with a current dose of 48 mg/day. She was also diagnosed with type 2 diabetes mellitus 6 years ago, with poorly controlled glucose levels and a recent HbA1c of 8.4% (normal range, 4–5.6), currently being treated with 20 units of insulin per day.

Twenty days before the presentation, the patient experienced persistent, moderate epigastric pain, along with frequent episodes of non-bloody, loose stools—occurring 10 to 20 times per day. These symptoms were accompanied by nausea, loss of appetite, and a significant weight loss of 20 kilograms over the course of 1 month. She denied any fever, night sweats, dysuria, and abnormal discharge. She was admitted to a local hospital and received standard treatment for gastritis with proton-pump inhibitors (PPIs) and fluid and electrolyte replacement; however, her condition did not improve. Therefore, she was transferred to our hospital.

On admission, she was alert, fatigued, afebrile, and in moderate distress, with a heart rate of 110 bpm, blood pressure of 90/60 mmHg, respiratory rate of 20 bpm, and a BMI of 18 with 45 kg. The physical examination revealed moderate epigastric tenderness without notable signs, abdominal distension, and no hepatosplenomegaly. She also had a Cushing appearance and conjunctival pallor. Initial laboratory tests showed moderate normocytic anemia with a hemoglobin level of 96 g/L (normal range, 130–170), mean corpuscular volume of 89 FL (normal range, 79–97), leukocytosis with white blood cell count of 14.69 G/L (normal range, 4–10), neutrophil count of 10.5 G/L (normal range, 1.9–8), eosinophil count of 0.12 G/L (normal range, 0–0.8), severe hypoalbuminemia with a level of 14 g/L (normal range, 35–52), severe hypoglycemia with a level of 1.34 mmol/L (normal range, 4.1–5.6), creatinine level of 29 umol/L (normal range, 59–104), sodium levels of 130 mmol/L (normal range, 135–145), potassium levels of 3 mmol/L (normal range, 3.5–5.0), lipase level of 50 UI/L (normal range, <67), C-reactive protein level of 34 mg/L (normal range, 0–5), and 24 h urine protein level of 0.5 g/L (normal range, <0.1). Fecal microscopy for parasites was negative, and fecal polymerase chain reaction (PCR) for bacteria was negative. Abdominal ultrasound was indicated and revealed unremarkable findings. The patient was treated with fluid administration, glucose infusion, pain control, intravenous cephalosporin antibiotics, and electrolyte management.

**Figure 1:**
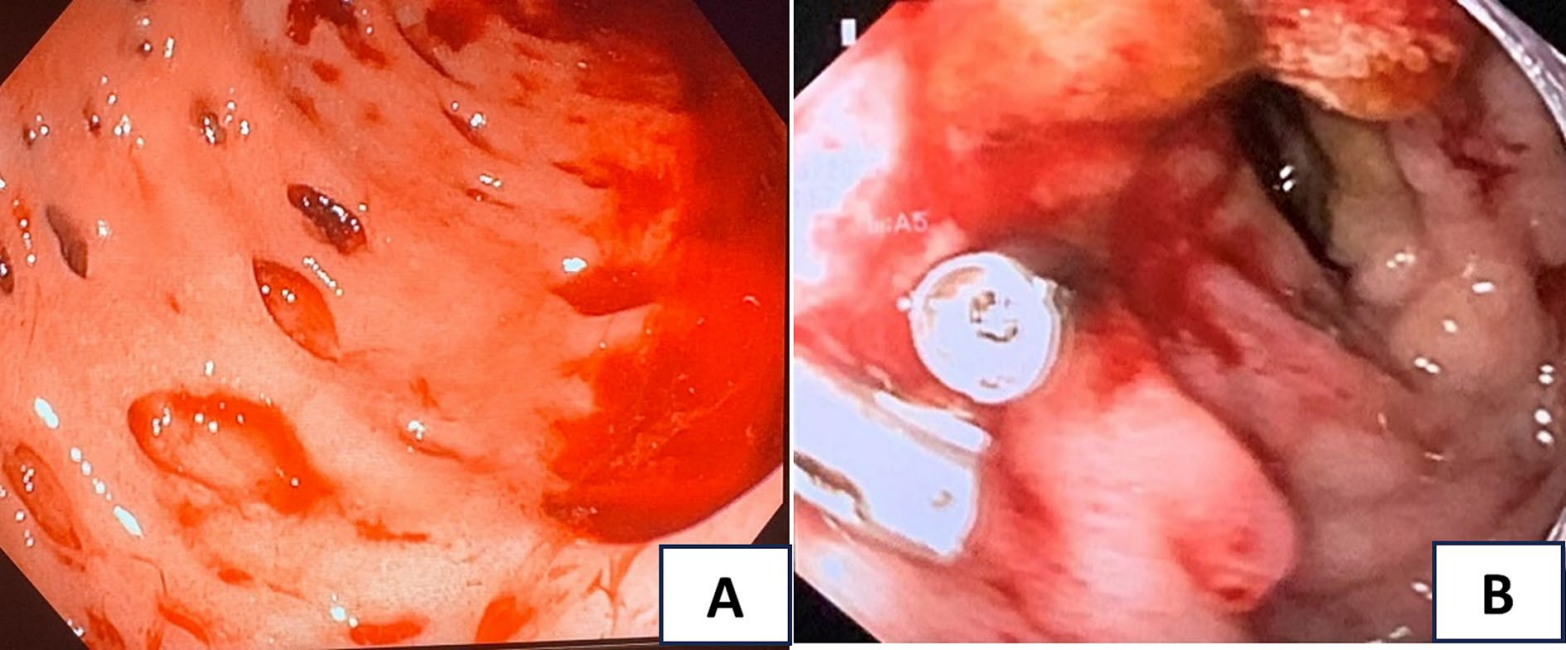
(A) The first upper endoscopy showed multiple actively bleeding duodenal ulcers with finger-shaped appearance; (B) the second upper endoscopy revealed active ulcers in the duodenum clipped.

**Figure 2:**
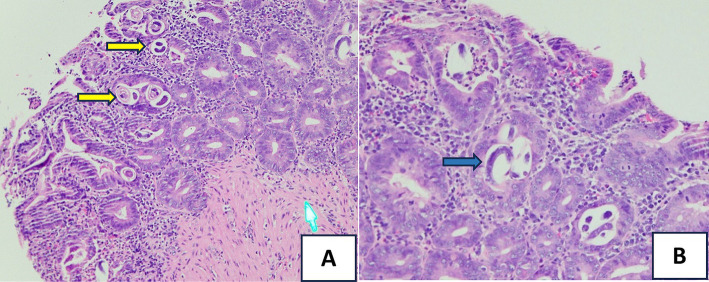
Hematoxylin and eosin (H&E) stain of the small bowel biopsy: (A) showing *S. stercoralis* eggs and larvae (the yellow arrows, HE x 20) and (B) revealing *Strongyloides* organisms (the blue arrow, HEx40) within the intestinal crypts.

On day 2 of admission, the patient suddenly had a large amount of black, tarry stools with stable hemodynamics and a significant hemoglobin drop from 96 to 68 g/L. The upper GI endoscopy was performed and showed multiple actively bleeding duodenal ulcers with finger-shaped appearance (Figure 1A), which were biopsied. The patient was treated with IV continuous PPIs, blood transfusion, and parental nutrition support. On day 3, the patient still had epigastric pain and severe melena; therefore, the second upper endoscopy was performed and clipped two active ulcers in the duodenum (Figure 1B). The repeated fecal microscopy was negative for parasites, pathological results revealed *Strongyloides stercoralis* eggs and larvae (Figure 2), PCR analysis of a duodenal sample confirmed CMV infection, plasma IgG for CMV was positive, and blood PCR for CMV was negative. An immunohistochemical stain for CMV was not available at our hospital at this time. The patient received albendazole 400 mg/day and IV ganciclovir 500 mg/day. The third endoscopic intervention was performed due to persistent severe melena, which revealed a Forrest IB ulcer at the duodenum, which was treated with Hemospray and clipping to stop the bleeding. Blood glucose levels were tested once daily, ranging from 9 to 14 mmol/L.

On day 5, despite optimal IV PPI therapy, the patient experienced severe melena, leading to hypotension with a blood pressure of 70/40 mmHg and tachycardia, lethargy, and hemoglobin levels dropping to 56 g/L. Blood gas showed metabolic acidosis with values of pH 7.15, bicarbonate level of 3.8 mmol/L, lactate level of 1.0 mmol/L, and severe hyperglycemia with glucose levels of 27.8 mmol/L. Urinalysis showed a 3+ ketone body. The patient was diagnosed with hypovolemic shock and diabetic ketoacidosis (DKA) and transferred to the intensive care unit (ICU). The patient was treated with fluid resuscitation, vasopressor therapy, glycemic management, blood transfusions, and continuous high-dose PPI therapy.

**Figure 3:**
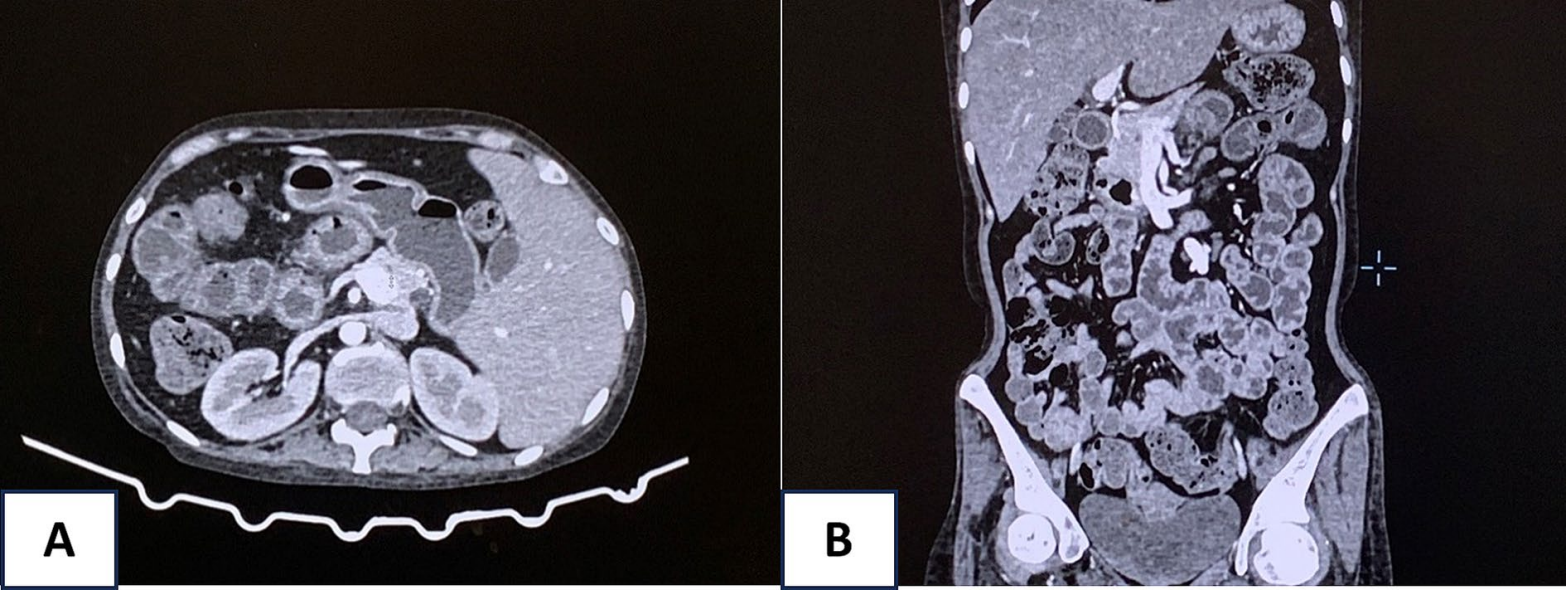
Abdominal computed tomography (CT) scan revealed edema and a thickening wall throughout the intestinal loops from the duodenum downward (A, axial view and B, coronal view).

Despite aggressive interventions, the patient’s condition remained unstable, with persistent hematochezia. An abdominal computed tomography (CT) scan revealed edema and a thickening wall throughout the intestinal loops from the duodenum downward, indicating potential bleeding from multiple sites in the digestive tract (Figure 3). Surgical interventions and gastroduodenal embolization were considered but deemed highly risky due to the patient’s critical condition and multiple bleeding locations. After consulting with multiple specialists, a fourth endoscopic examination was performed, revealing active bleeding from duodenal ulcers. The duodenal ulcers were found to be inflamed, edematous, and congested, with a propensity to bleed upon contact. Bleeding control was achieved using clipping and Hemospray. The patient received ivermectin at a dose of 0.2 mg/kg with 9 mg once daily.

**Figure 4:**
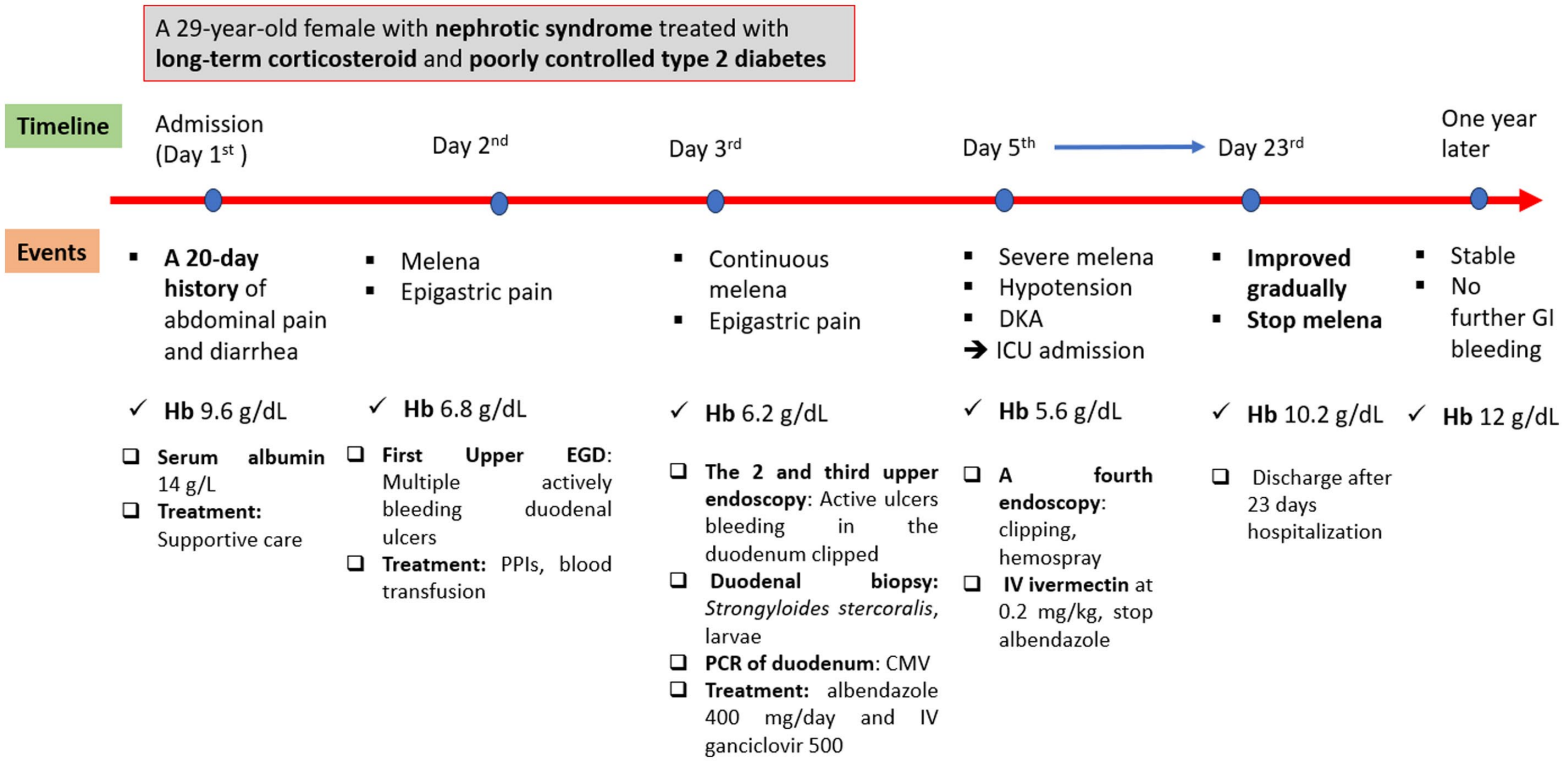
Events of the patients.

The patient’s condition improved gradually, and she was then transferred to the gastroenterology department after 3 days in the ICU. She continued to use ivermectin at 9 mg once daily for 14 days (due to her critically severe condition) and ganciclovir at 500 mg/day intravenously, *Pneumocystis jirovecii* pneumonia prophylaxis with trimethoprim–sulfamethoxazole, and nutritional support. She was discharged after 23 days at the hospital. At a 1-year follow-up, the patient remained in stable condition. Figure 4 reveals the events of this patient.

Written informed consent was obtained from the patient for publication of this case report and any accompanying images.

## 3 Discussion

Cytomegalovirus infection of the GI system is a major cause of morbidity and mortality in immunocompromised patients (1). Following primary infection, CMV establishes latency within host tissues, existing in equilibrium with the immune system. Reactivation of latent CMV, primarily driven by immunosuppression, frequently leads to infection of the gastrointestinal tract, while superinfection in the setting of pre-existing GI diseases, such as inflammatory bowel disease, is less common (10). While CMV can infect any segment of the digestive system, from the esophagus to the rectum, the colon represents the most frequent site (55%), followed by the esophagus and stomach (40%), with the small intestine being rarely involved (4.3%) (11). Our case had the predominant lesions on the duodenum and early part of the small intestine based on imaging findings. CMV targets vascular endothelial cells and surface epithelial cells within the small intestine, inducing submucosal hemorrhages, erosions, and both superficial and deep ulcers. These pathologic changes manifest clinically as persistent diarrhea, weight loss, occult gastrointestinal bleeding, intestinal obstruction, perforation, and, infrequently, massive GI bleeding (12). Colonoscopy identified a spectrum of characteristic mucosal abnormalities in patients with CMV-associated colitis. These included deep ulcers, manifesting as profoundly excavated lesions approaching or surpassing the muscularis propria layer, with or without mildly elevated margins. Punched-out ulcers were also observed, characterized by their near-circular shape and distinct demarcation (13). Our case had a negative result of blood CMV PCR. The sensitivity of CMV PCR varies between 81 and 87%. It is essential to note that whole blood CMV PCR exhibits a positive predictive value as low as 34% for tissue-invasive CMV disease (3). Although CMV immunohistochemical staining (the gold standard for diagnosing tissue-invasive disease) was not performed in our case, the presence of multiple characteristic punched-out ulcers strongly suggested CMV infection. This was further supported by positive plasma IgG, mucosal PCR for CMV, and clinical findings, which collectively indicated a localized CMV infection within the gastrointestinal tract, with no evidence of systemic involvement at the time of testing.

The nematode *Strongyloides stercoralis* causes strongyloidiasis and encompasses a heightened concentration in tropical regions characterized by warm temperatures, high humidity, and inadequate sanitation (4). In Vietnam, its presence was first documented in 1876 among French soldiers suffering from severe diarrhea, later establishing its status as endemic. Subsequent meta-analyses using stool and serological examinations revealed diverse prevalence estimates, ranging from 0.2 to 2.5% in the north and 1.19 to 7.6% in the south, respectively (14). The unique life cycle of *S. stercoralis* distinguishes it from other human parasites due to its capacity for autoinfection. Gastrointestinal manifestations often include nausea, vomiting, malabsorption, abdominal discomfort, and melena (5). The diagnostic method involves using microscopy to identify *S. stercoralis* larvae in stool samples directly. However, the sensitivity is quite low, with only approximately 21%, due to the intermittently excreted larvae and low numbers, with larvae not always being shed sufficiently to be detected in a single sample and often requiring multiple examinations. Some studies show that sensitivity improves to 50% with three stool samples and can approach 100% with seven consecutive samples (15, 16). Endoscopy with a sample biopsy from the GI tract can make the diagnosis. Accelerated autoinfection involves a significant increase in larval migration within the organs and may result in disseminated disease outside the GI tract. Corticosteroid usage is the primary risk factor for hyperinfection in the developed world (17). In our case, the stool test was negative, ruling out hyperinfection. However, the Strongyloidiasis may exacerbate gastrointestinal symptoms, particularly contributing to malabsorption with severe hypoalbuminemia, worsening gastrointestinal bleeding, and impaired healing of duodenal ulcers. Therefore, we should perform more consecutive samples to increase the possibility of detecting *S. stercoralis*.

When assessing ulcerative lesions in the GI tract of an immunocompromised patient, the macroscopic features of ulcers can provide valuable diagnostic clues about various pathogens. Our patients had multiple actively bleeding duodenal ulcers with a finger-shaped, punched-out appearance, which is typically for CMV, while endoscopic findings of *S. stercoralis* on duodenum often included brown discoloration, edema, erythema of mucosa, rarely with shallow, small ulcers (only 2 out of 25 cases) that are prone to bleeding (18). Herpes simplex virus ulcers are typically multiple small shallow, well-circumscribed, with raised edges and vesicles (19), while candida infections often present as white plaques with underlying shallow ulcers (20), and *Mycobacterium tuberculosis* can cause shallow, circumferential, or transverse ulcers with irregular margins (21). Moreover, these pathogens rarely cause multiple ulcers in the duodenum. Therefore, they were less likely to result in these lesions. These features help guide the differential diagnosis, although confirmation requires histopathological and microbiological testing.

Our case represented a unique condition of coinfection of CMV and *S. stercoralis* in an immunocompromised patient complicated with hypovolemic shock caused by GI bleeding and DKA. This patient, who had poorly controlled type 2 diabetes and long-term corticosteroid use, was considered immunocompromised, which is the risk factor for both CMV and *S. stercoralis* infection. Compared to a review of 21 case reports, the median age of 47 years was quite higher than our case, primarily from the United States or India, with only one case from Asia and only 9.5% of patients having diabetes mellitus (22). In our case, we thought that CMV plays a key role in GI bleeding because the patient’s presentation was predominant for GI bleeding, and typical endoscopic findings suggested CMV while *S. stercoralis* facilitated this condition. Other cases of *Strongyloides* and CMV coinfection often happened in patients with solid organ transplantation or hematologic diseases, which resulted in hyperinfection syndrome caused by *S. Stercoralis* and combined involvements of GI, pulmonary, and viremia (22, 23). However, coinfection cases with only GI symptoms and GI bleeding are rarely reported. To the best of our knowledge, there are only two cases of this coinfection causing mild GI bleeding (7, 8), and our case is the first case presenting with severe hypovolemic shock and DKA. To prevent severe complications like our case, it is critical to make an early diagnosis and promptly give the treatment. CMV tests should be screened in patients with typical endoscopic findings, immunosuppression, or transplant history. Coinfection with *S. stercoralis* should also be considered with caution, particularly when no other clear etiology for the symptoms is identified, given the risk of *S. stercoralis* exacerbation leading to severe hyperinfection (7, 17).

Our patient had a challenging treatment due to persistent GI bleeding. The patient had undergone four endoscopies with difficulty in controlling bleeding, which can be explained by the multiple lesions distributed in both the duodenum and the small intestine that could not intervene with endoscopy. Furthermore, the coinfection of CMV and *S. stercoralis* caused deep ulcers and fragile mucosa, which made it difficult for endoscopic mechanical interventions. Our patient was initially treated with albendazole due to ivermectin not being available in our hospital. In a meta-analysis, the efficacy of ivermectin has been shown to be greater than that of albendazole (24). Due to the severe conditions of the patient, we tried to switch to ivermectin for 14 days, which is not the standard duration of care; however, the patient tolerated the treatment well and improved symptoms rapidly. A review showed that ivermectin was used in 11 cases (66.7%), and the mortality rate of this coinfection was still high (52.4%). However, no cases with GI symptoms had a hemorrhage shock and needed multiple GI interventions to control GI bleeding (22).

## 4 Conclusion

This case highlights the importance of considering the possibility of coinfection with CMV and *S. stercoralis* in immunocompromised patients presenting with gastrointestinal symptoms, especially in those with a long-term history of corticosteroid use. Early recognition of both pathogens is crucial as timely diagnosis and treatment can prevent severe complications. Physicians should be suspicious and screen for this coinfection, particularly in patients with immunocompromised conditions or unexplained gastrointestinal bleeding and systemic symptoms. Prompt initiation of appropriate therapy, including antiparasitic and antiviral agents, can significantly improve patient outcomes in these groups.

## Data Availability

The original contributions presented in the study are included in the article/Supplementary material, further inquiries can be directed to the corresponding author.
